# Atmosphere Effects in Laser Powder Bed Fusion: A Review

**DOI:** 10.3390/ma17225549

**Published:** 2024-11-13

**Authors:** Ben Brown, Cody Lough, Davis Wilson, Joseph Newkirk, Frank Liou

**Affiliations:** 1 Materials Engineering, Department of Energy’s Kansas City National Security Campus, Kansas City, MO 64147, USA; 2 Welding Engineering, Department of Energy’s Kansas City National Security Campus, Kansas City, MO 64147, USA; davis.wilson@framatome.com; 3 Nuclear Engineering and Radiation Science Department, Missouri University of Science and Technology, Rolla, MO 65401, USA; 4 Mechanical and Aerospace Engineering Department, Missouri University of Science and Technology, Rolla, MO 65401, USA

**Keywords:** additive manufacturing, laser powder bed fusion, cover gas, pressure

## Abstract

The use of components fabricated by laser powder bed fusion (LPBF) requires the development of processing parameters that can produce high-quality material. Manipulating the most commonly identified critical build parameters (e.g., laser power, laser scan speed, and layer thickness) on LPBF equipment can generate acceptable parts for established materials and moderately intricate part geometries. The need to fabricate increasingly complex parts from unique materials drives the limited research into LPBF process control using underutilized parameters, such as atmosphere composition and pressure. As presented in this review, manipulating atmosphere composition and pressure in laser beam welding has been shown to expand processing windows and produce higher-quality welds. The similarities between laser beam welding and laser-based AM processes suggest that this atmosphere control research could be effectively adapted for LPBF, an area that has not been widely explored. Tailoring this research for LPBF has significant potential to reveal novel processing regimes. This review presents the current state of the art in atmosphere research for laser beam welding and LPBF, with a focus on studies exploring cover gas composition and pressure, and concludes with an outlook on future LPBF atmosphere control systems.

## 1. Introduction

In recent years, there has been an increasing push to adopt end-use components produced by additive manufacturing (AM) in high-consequence applications such as those in the aerospace industry, as detailed by Liu et al. [1]. Of the commercially available AM technologies, laser powder bed fusion (LPBF) has garnered significant attention due to its ability to produce high-quality components with higher detail when compared to other AM methods, such as electron beam powder bed fusion (EBPBF) [2] or laser-directed energy deposition (LDED) [3]. Successful adoption of this technology requires users to develop a process that utilizes the LPBF machine’s settings in a way that reliably produces high-quality material that meets the end application’s needs. This optimization currently does not generally include manipulating the atmosphere and is typically limited to the number of critical build parameters such as laser power, laser scan speed, laser hatch pattern, layer thickness, and laser focus [4]. As will be shown in this review, manipulating the atmosphere in addition to laser beam parameters is common in welding research and has significant effects on processing. As a result, the literature presented in this review is primarily derived from applicable welding research to demonstrate the utility of atmosphere manipulation. However, Section 5 presents the limited current state of the art of atmosphere manipulation in LPBF.

As described by Oliveira et al. [5], because of the numerous similarities in fundamental concepts, much can be applied from the welding community’s research on processing parameter development to laser-based AM. A review of processing parameters by Oliverira et al. [4] suggests that benefits beyond merely preventing oxidation can be realized through the manipulation of the atmosphere. A review by Shi et al. [6] on vaporization in LPBF highlights the advantages of varying atmospheric conditions, but also notes the limited amount of literature on the subject.

There are key differences, however, that must be considered when comparing these methods. Laser beam welding, compared to LPBF, generally uses laser powers greater than 1 kW, whereas LPBF laser powers are typically at or below 500 W for most commercially available equipment. Importantly, the smaller laser spot sizes in LPBF result in power densities that are on the same order of $10^{6}$ W cm^−2^ [7], as typically seen in laser beam welding, as demonstrated by the experimental setup used by Gao et al. [8]. For this reason, comparisons to the welding literature, despite differing laser setups, are still applicable. In the welding literature surveyed for this review, several different types of lasers with various wavelengths, resulting from their distinct constructions, were used, including CO_2_ (λ = 10.6 μm), Nd:YAG (λ = 1064 nm), and Yb-fiber (λ = 1070 nm). Although diode lasers or gas tungsten arcs were used less frequently, literature involving these types is also considered relevant. While the Yb-fiber laser is exclusively used in modern LPBF equipment, research utilizing these other laser types was considered and will be noted when results are presented.

To provide context on how the manipulation of atmospheric composition in Section 3 and pressure in Section 4 affect processing and properties, the following provides additional details on defect generation and laser beam attenuation in laser-based processes.

## 2. Mechanisms of Defect Generation and Laser Beam Attenuation

**Figure 1:**
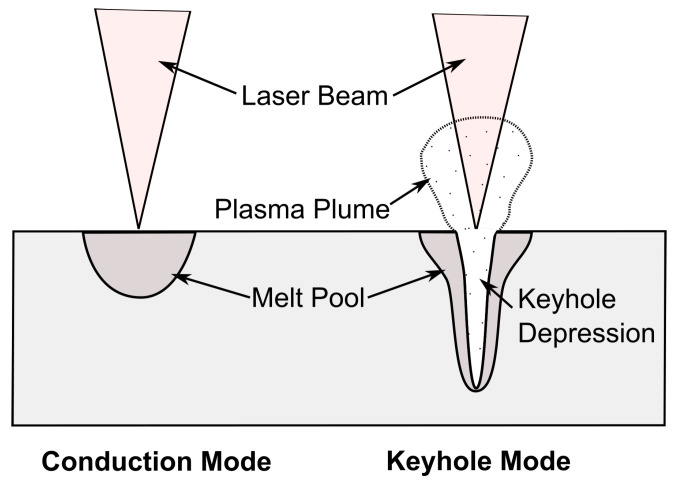
Schematic comparing the differences between conduction and keyhole mode melting.

During laser–metal interactions, as laser energy increases, the mode of melting progresses from conduction mode to keyhole mode. In conduction mode, the laser only interacts with the liquid at the surface of the melt pool via direct absorption, where less than 30% of the laser energy is typically absorbed [9]. When enough laser energy is applied, the liquid in the melt pool will begin to vaporize and the pressure of this metal vapor being generated pushes down into the liquid, resulting in the formation of a localized depression. The increased surface area of the depression allows for the laser energy to be absorbed both on the surface of the melt pool and along the walls of the depression. The added surface area enables additional instances of Fresnel absorption via reflection in the depression as well as the interaction with the metal vapor in and above the depression. These additional vectors for energy absorption result in a more efficiently coupled laser beam, where as much as 90% of the laser energy is absorbed by the melt pool [9]. This mode of melting—where a depression forms, allowing for more energy to be absorbed—is known as keyhole mode melting due to the high depth-to-width ratio and the weld shape’s resemblance to a lock’s “keyhole”. The differences between the conduction and keyhole mode melting are illustrated in Figure 1.

**Figure 2:**
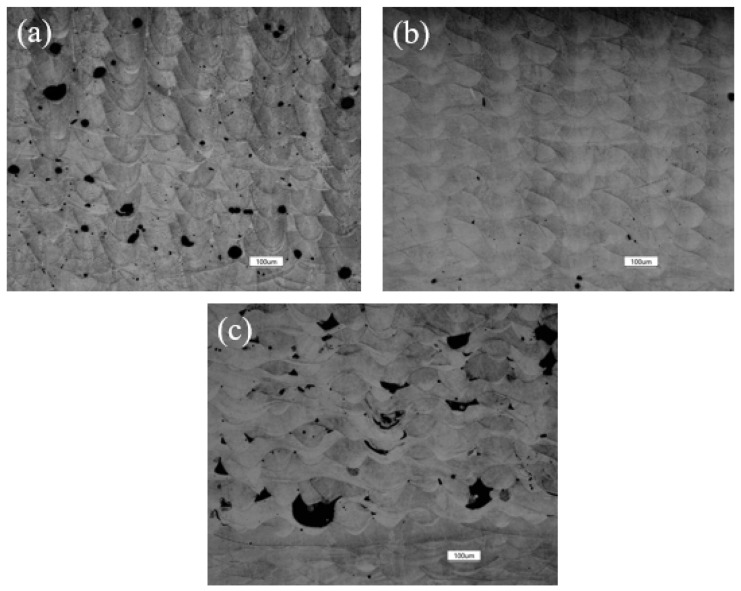
Micrographs of LPBF Inconel 625 with varying parameters showing different melt pool geometries and defect types. (**a**) Demonstrates keyhole mode melting and resulting porosity. (**b**) Demonstrates a more nominal conduction mode melting with some stochastic porosity still present. (**c**) Demonstrates conduction mode melting with insufficient energy, resulting in a lack of fusion-type porosity. Reproduced with permission from [12]. 2022, Elsevier.

**Figure 3:**
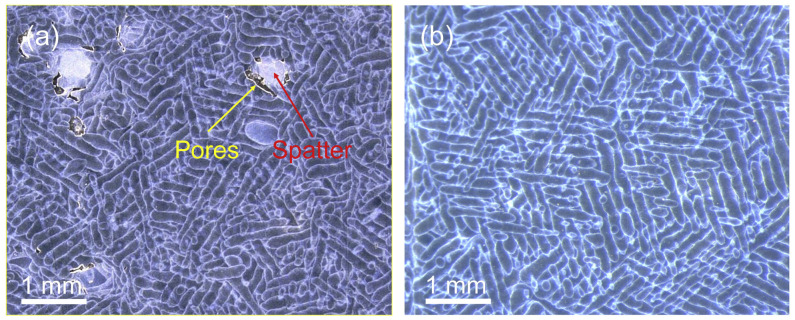
Micrographs displaying an example of excess spatter and the resulting porosity in LPBF AlSi10Mg. The sample shown in (**a**) was built with a 50 μm layer and the sample in (**b**) was built with a 30 μm layer. Reproduced with permission from [13]. 2018, Elsevier.

As the laser energy increases and the resulting depression increases in size, the local pressure balance of metal vapor and liquid forces within the melt pool can become unbalanced, leading to cavity collapse. An unstable melt pool cavity can result in the entrapment of gas bubbles [10] and contribute to increased spatter ejection [11], both of which result in porosity. Examples of keyhole and spatter porosity in LPBF material are shown in Figure 2 and Figure 3, respectively.

The metal vapor along with the surrounding cover gas interacts with the laser and can be ionized to form a plasma plume of metal vapor and cover gas [14]. Interactions between the laser heat source and plume are dependent on the laser wavelength, the power of the laser, the ionization energy of the metallic vapor, and the ionization energy of the cover gas. Metal vapors are more easily ionized than the common cover gases listed in Table 1, whereas iron vapor ionizes at 7.9 eV, titanium vapor at 6.8 eV, and aluminum vapor at 6.0 eV [15]. Plumes over the melt pool attenuate the laser beam, reducing the amount of laser energy delivered.

There are three main mechanisms for melt pool plume attenuation of the laser beam: the electron–photon interaction, known as inverse bremsstrahlung absorption, the diffraction phenomenon, called Rayleigh scattering, which occurs for beams of wavelengths much larger than the interacting particles; and the refraction of the beam as it passes through the plume, resulting in a larger effective spot size [19]. As will be shown in this review, atmosphere manipulation can be an effective means to reduce these modes of attenuation, resulting in fewer defects.

The inverse bremsstrahlung absorption coefficient, $K_{a}$, as described by Matsunawa and Kim [20], can be calculated by Equation (1), where $n_{e}$ denotes electron number density, $n_{i}$ denotes ion number density, *z* denotes the charge number, *e* denotes the electronic charge, *C* denotes the velocity of light, $\epsilon_{0}$ denotes the dielectric constant of the medium, $m_{e}$ denotes the mass of electron, $k_{B}$ denotes the Boltzmann constant, *T* denotes temperature, ω denotes angular frequency of the laser beam, $\omega_{pe}$ denotes the angular frequency of plasma oscillation, and lnΛ denotes the Coulomb logarithm.

(1) $$K_{a}=\frac{z^{2}e^{6}n_{e}n_{i}ln\Lambda }{3\omega^{2}C\epsilon_{0}^{3}{\left(2\pi m_{e}k_{B}T\right)}^{\frac{3}{2}}\sqrt{1-{\left(\frac{\omega_{pe}}{\omega }\right)}^{2}}},$$

The Rayleigh scattering coefficient $K_{sca}$, as described by Matsunawa and Kim [20], can be calculated by Equation (2), where *N* denotes the number density of the particle, *V* denotes particle volume, ϵ denotes the dielectric constant of the particle, $\epsilon_{0}$ denotes the dielectric constant of the medium, and λ denotes the wavelength of the incident beam.

(2) $$K_{sca}=\frac{8\pi^{3}NV^{2}}{3\lambda^{4}}{\left(\frac{\epsilon -\epsilon_{0}}{\epsilon_{0}}\right)}^{2},$$

At longer laser wavelengths, like that of a CO_2_ laser, attenuation is primarily a result of inverse bremsstrahlung absorption, as $K_{a}$ is proportional to the square of the laser wavelength and both metal vapor and cover gas plasmas can be observed [21]. At shorter laser wavelengths of a Yb-fiber laser, Rayleigh scattering via ultra-fine condensate particles dominates, as this attenuation $K_{sca}$ is inversely proportional to the fourth power of the beam wavelength [20], and emissions from only the metal vapor are observed in welding [8] and LPBF [22]. As a result of the different laser wavelengths, the plasma plume temperature of a CO_2_ laser will be on the order of 8000–10,000 K [23], whereas a Yb-fiber laser-generated plume will be much cooler and range from 4000 to 6000 K [24]. The formation of excessive plasma plumes over the melt pools can significantly reduce the effective laser energy delivered to the work surface. In the LPBF application, this reduced delivered laser energy can lead to a lack of fusion pores, as seen in Figure 2c.

### Section Summary and Impact on LPBF

The amount of laser power delivered to a work surface is reduced by interaction with the generated plasma plume. This occurs through attenuation via inverse bremsstrahlung absorption and Rayleigh scattering, as well as plume refraction, resulting in a larger effective spot size and lower power density. In LPBF applications, reduced laser beam attenuation enables more efficient use of the limited laser power available in commercial equipment. The reduction in plume refraction—in addition to improving power density at the work surface—provides additional constancy throughout the build of the part. Reduced attenuation and improved stability allow for better tuning of process parameters, which helps avoid defects associated with excessive energy, such as keyhole porosity and spatter, as well as defects due to energy defects, like lack of fusion.

## 3. Atmosphere Composition Effects in Laser Beam Welding

In laser beam welding, inert gases, reactive gases, and mixtures of the two are used as cover gases, depending on the application. Inert gases (argon and helium) as well as reactive gases (nitrogen and carbon dioxide) are most commonly used in laser beam welding [25]. These gases have different properties and interact differently when used as cover gases, resulting in different melt pool characteristics. A summary of properties for these common gases can be found in Table 1. Of the inert gases, helium has a higher thermal conductivity, heat capacity, and ionization energy than argon. Lower than helium, nitrogen also has a higher thermal conductivity and heat capacity than argon. Nitrogen—as a diatomic molecule—can disassociate into monatomic nitrogen, given sufficient energy. Carbon dioxide has various dissociations as well that can occur where carbon monoxide, oxygen, and carbon are formed. In LPBF, pure argon is currently the predominant choice for cover gas due to its availability and inert nature, allowing it to be used for a wide variety of materials. While nitrogen is occasionally used, its application is limited due to its reactivity.

### 3.1. Helium

As previously outlined, the presence of a plasma plume over the melt pool will attenuate the laser power delivered to the work surface. One method of reducing plasma plume formation and increasing the delivered laser power, as demonstrated in the welding literature, involves substituting helium into the cover gas mixture. As detailed in Table 1, helium has higher ionization energy, thermal conductivity, and heat capacity than both argon and nitrogen. Numerous studies using both long and short-wavelength lasers have shown that—by this substitution—deeper melt pools are produced. Shanmugrajan et al. [26] demonstrated higher depths of penetration in P92 steel welds when using a CO_2_ laser compared to the use of argon or argon-nitrogen atmosphere. Using a Yb-fiber laser, Ahn et al. [27] showed that autogenous welds under helium of 2024-T3 aluminum were hotter, and as a result, keyhole porosity had more time to escape before the melt pool solidified than welds produced under argon at the same laser settings. Cai et al. [28], when using a hybrid Yb-fiber laser-arc setup, showed that helium additions to an argon cover gas reduced porosity and increased weld depth for aluminum welds proportionally, with additions of up to 50% by volume.

**Figure 4:**
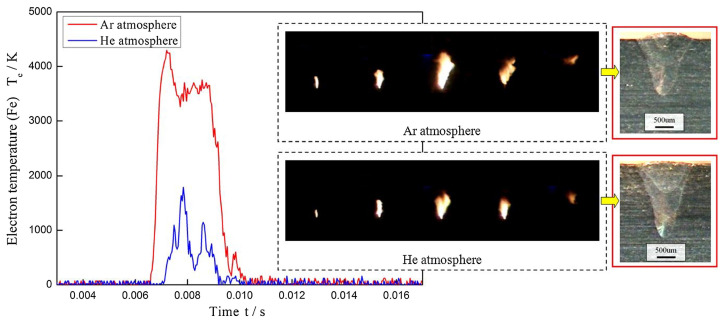
Observed results of reduction in electron temperature and plasma plume under helium cover gas compared to argon when using pulsed Nd:YAG laser-welded 304 stainless steel. Increased penetration depth is also observed as a result of helium cover gas. Reproduced with permission from [30]. 2019, Elsevier.

When used with Nd or Yb-fiber lasers, where a cover gas plasma is not generated, helium’s high thermal conductivity and heat capacity are the primary factors in reducing attenuation by cooling the plasma plume. Attributing these results to helium’s ability to produce a cooler plasma, which has lower laser beam attenuation, is in line with the results presented by Giacobbe [29], where the heat transfer behavior of pure helium was shown to be superior at low and high flow rates compared to argon and nitrogen. Giacobbe also showed that, at intermediate flow rates around the transition from laminar to turbulent flow, binary mixtures of helium–argon and helium–nitrogen can outperform pure helium. Xu et al. [30] measured plume temperature for Nd:YAG laser beam welds of steel and determined that substituting helium into argon decreased overall plume temperature, reducing the calculated inverse bremsstrahlung absorption coefficient. The results shown in Figure 4 demonstrate how the use of helium reduces overall laser beam attenuation through the reduction of plume temperature and relative density per Equation (1). With a reduced plume, the resulting penetration depth is increased. Motlagh et al. [31] presented a similar result with Nd:YAG lasers, with the highest penetration depths observed in pure helium cover gas over argon. As expected by the principals laid out in Section 2, a significant increase in inverse bremsstrahlung absorption was shown when CO_2_ lasers were used.

The beneficial effect of plume reduction is enhanced with an increased cover gas flow rate that results in increased heat transfer away from the plume. Dai et al. [32] measured the decreasing plasma temperature from magnesium CO_2_ laser beam welds with increasing helium flow rate. Sathiya et al. [33] observed welds with finer microstructures compared to argon and nitrogen atmosphere, potentially a result of faster cooling in the melt pool. This conclusion agrees with the observations of LaCroix et al. [34], who noted that when substituting nitrogen into argon—where nitrogen has higher thermal conductivity and heat capacity—plasma plume temperatures were reduced.

### 3.2. Nitrogen

The generation of a hot plasma plume can result in increased absorption of reactive gases through the dissociation of gas molecules and the increased availability of more easily absorbed monotonic gas species [21]. When nitrogen cover gas is used, increases in base material nitrogen have been measured. Dong et al. [14] demonstrated a reduction in nitrogen absorbed into iron-based alloys when going from an arc heat source to a CO_2_ laser, to a Nd:YAG laser, with reduced plume temperature resulting in less monatomic nitrogen available to be absorbed. Wu et al. [35] compared the nitrogen effects of a CO_2_ laser and a Yb-fiber laser beam welding of 301L austenitic steel and did not observe any major differences between the use of the two laser types. The absorbed nitrogen in this study was found to be concentrated at the surface of the melt pools in the form of MN nitrites. Hafez et al. [36] showed nitrogen pickup in Yb-fiber laser beam welds of 304 steel in quantities proportional to the concentration of added nitrogen in the cover gas. This increase in nitrogen concentration also reduced the amount of delta ferrite in the final material. Shanmugaraja et al. [26] noted reduced delta ferrite in P92 welds, as well as the absorbed nitrogen for the cover gas. Lai et al. [37] observed increased amounts of austenite after Yb-fiber laser beam welding of duplex steel with nitrogen added to the argon cover gas. They attributed this effect to nitrogen uptake from the cover gas, which stabilizes the austenite phase. Similarly, Keskitalo et al. reported reduced austenite when welding duplex steel under an argon atmosphere [38]. Zhang et al. [39] measured increased hardness in CO_2_ laser beam welding of duplex steel when using nitrogen substitutions in argon cover gas. In pulse processing of Vitreloy 1 metallic glass, Huang et al. [40] observed cracking due to ZrN formation when processing under a nitrogen cover gas. They noted that the desorption of nitrogen from the nitrogen-containing base material decreased when welding in nitrogen environments. A similar effect was reported by Dong et al. [41] during CO_2_ laser beam welding of high nitrogen steels.

Numerous studies have shown that the solubility and reactivity of nitrogen in the base material can have a direct effect on material microstructure and properties. The reactivity of nitrogen gives it the benefit of increased absorption of gas pores trapped in the melt pool, leaving a higher-density solidified material compared to welds under argon. Zhao et al. [42] concluded that CO_2_ laser beam welded high nitrogen steel would absorb nitrogen into the base metal and produce welds with fewer pores when exposed to increasing quantities of nitrogen in the cover gas. Katayama et al. [43] demonstrated a reduction in weld porosity with the use of nitrogen cover gas while exploring the relationship between scan speed, laser power, and their effects on entrapped gas bubbles. Elmer et al. [44] observed the near elimination of porosity in Yb-fiber welds of various steels under nitrogen cover gas. Sun et al. [45] reported a similar reduction in porosity in Yb-fiber welding of 340L stainless steel.

### 3.3. Carbon Dioxide and Oxygen

**Figure 5:**
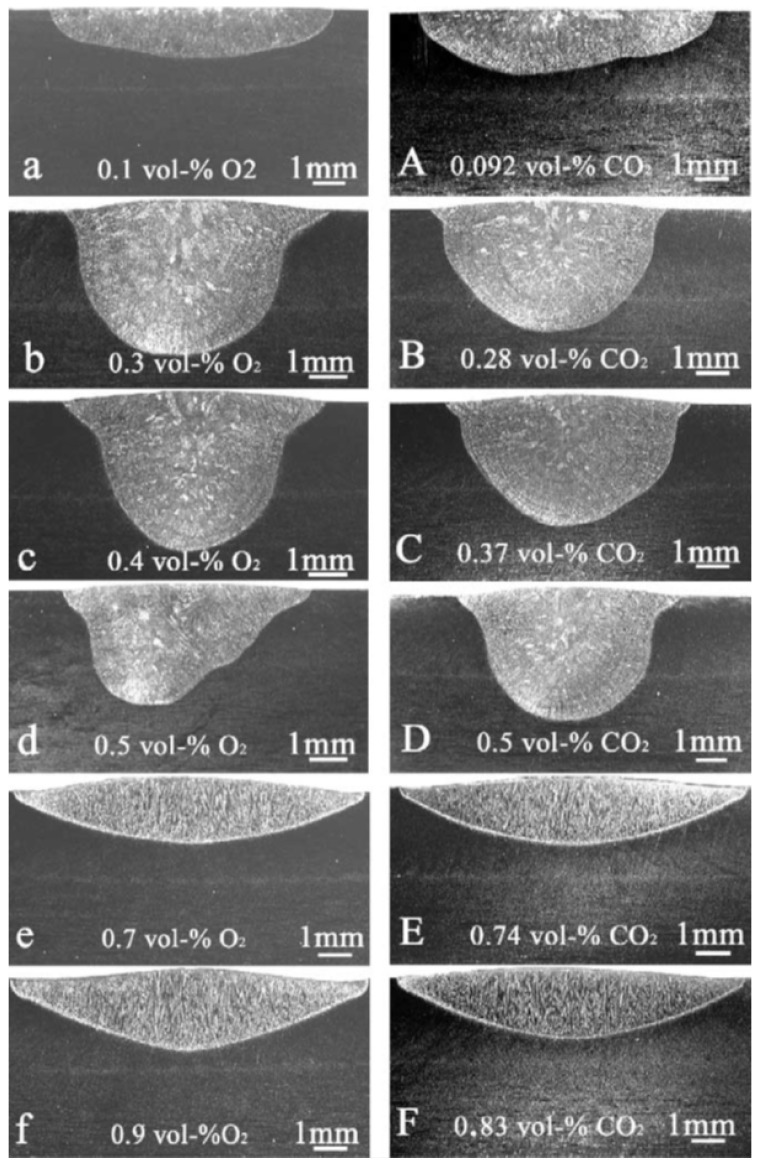
Evolving melt pool shape in gas tungsten arc welded 304 stainless steel under argon cover gas, affected by increasing levels of oxygen shown in (**a**–**f**) on the left, and increasing carbon dioxide shown in (**A**–**F**) on the right. Small amounts of oxygen and carbon dioxide promote the preferred Marangoni flow, enhancing melt pool depth (**b**–**d**,**B**–**D**). With sufficient addition, oxides begin to form, reverting the flow direction and causing shallow melt pools, as shown in (**e**–**f**,**E**–**F**). Reproduced with permission from [47]. 2004, Elsevier.

Similar to the effects observed with nitrogen cover gas, the interaction between the laser beam and plume can cause the dissociation of carbon dioxide molecules, leading to a range of effects. As detailed in Table 1, the carbon dioxide molecule can break down into combinations of carbon monoxide, oxygen, and carbon. Abbott et al. [18] showed that the quality of CO_2_ laser beam welds of mild steel with carbon dioxide cover gas is dependent on the deoxidizing potential of the base material. With sufficiently deoxidized steel via silicon additions, low-porosity welds can be made under carbon dioxide. Li et al. [46] observed oxide formation in Yb-fiber laser beam welds of 304L in pure carbon dioxide with fewer pores than welds produced under argon and argon–carbon dioxide. A melt pool shape difference was also observed with carbon dioxide-containing cover gas. The observed oxygen pickup and changes in the melt pool shape can be explained by the effect oxygen has on the surface tension of liquid metal. Lu et al. [47] characterized the impact of additional oxygen pickup in gas tungsten arc welds and its influence on the melt pool shape. As presented by Lu et al., as oxygen or carbon dioxide concentration in the cover gas increases, the oxygen in the liquid melt pool also increases. As a result, the Marangoni flow will change direction at a critical value, resulting in wider or deeper melt pools, depending on the flow direction. Low amounts of absorbed oxygen surface tension can be manipulated to shape melt pools or affect mechanical performance. If too much is absorbed, oxides can form, resulting in undesirable properties. This effect is demonstrated in Figure 5.

In addition to generated oxygen, some authors have evaluated adding portions of oxygen to the cover gas. In Nd:YAG laser beam welding of CP-Ti alpha phase morphology, Li et al. [48] observed changes and increased strength of up to 2% of oxygen substitution in argon. Brittle fracture occurred above 2% due to the continued evolution of alpha phase morphology. Boujha et al. [49] showed that the addition of oxygen in high-powered diode laser (λ = 940 nm & 808 nm) aluminum welds changed the melt pool shape as well as introduced oxides to the material.

### 3.4. Section Summary and Impact on LPBF

The purpose of a cover gas in a laser-based process is to isolate the melt pool from the surrounding environment to reduce contamination, such as excessive oxide formation, as well as remove the particulate from the laser beam’s path. The composition of the gas used will affect the process and the resulting material. With the use of longer wavelength lasers—where cover gas ionization can occur—the high ionization energy of helium reduces the plume generation and the corresponding laser beam attenuation. When paired with reactive gases, long-wavelength lasers increase the absorption of available species such as nitrogen or carbon dioxide, along with their breakdown constituents. This can have varying effects on material strength, phase amounts, and melt pool geometry.

With shorter wavelength lasers, like the Yb-fiber used in LPBF, similar effects are seen to those in the longer wavelength laser interactions. Helium reduces laser beam attenuation through cooling of the plasma plume, resulting in more laser power being delivered to the work surface. Although there is no direct interaction between the laser and the cover gas, absorption is still observed, resulting in the same material response seen in longer wavelength interactions, just at a reduced magnitude. Changes in phase composition, material strength, and melt pool shape are all still observed. When applied to LPBF, these attributes would enable expanded manipulation of the process.

## 4. Atmosphere Pressure Effects in Laser Beam Welding

Along with the composition of an atmosphere, as discussed in Section 3, the pressure at which these gases are used can have a significant effect on melt pool properties. The use of non-atmospheric pressures is less common in general for laser applications, as specialized equipment is needed in order to create a vacuum or a hyperbaric environment. There are, however, potential advantages in using non-atmospheric pressures. Jiang et al. [50] reviewed the current state of laser welding under vacuum, concluding that the primary benefits include improvements in plasma plume, keyhole, and melt pool behaviors, resulting in excellent welding performance. The ability to capture these effects through simulation has been recently demonstrated, as presented by Han et al. [51]. Recent advancements in non-atmospheric processing and content relevant to LPBF application will be presented in this section.

### 4.1. Vacuum

Attenuation via inverse bremsstrahlung absorption, Rayleigh scattering, and refraction is greatly reduced with the reduction in working pressure. At pressures below the atmospheric pressure of 101 kPa, it has been shown that the size and density of the plasma plume are reduced. This effect has been demonstrated across various laser beam types, powers, and work compositions, resulting in overall higher depths of penetration.

Assuming ideal gas behaviors for the plasma that forms over the melt pool during the laser–metal interaction, a reduction in pressure will reduce the total plume electron density $N_{e}$ calculated by the Sasha equation, shown as Equation (3) [52], where electron density $N_{e}$ is calculated by the following:

(3) $$N_{e}=2.16\times 10^{17}p^{1/2}T_{e}^{1/4}exp\left(\frac{-45.65\times 10^{3}}{T_{e}}\right),$$

where *p* denotes pressure and $T_{e}$ denotes electron temperature. As the electron density and gas density decreased, the attenuation coefficients for inverse bremsstrahlung absorption, as per Equation (1), reduced. In addition to reducing the electron–photon interactions, decreasing pressure also reduced the effect of the volume of the condensate generated, as shown by Matsunawa and Ohnawa [53]. A decreased volume of condensate generated will result in a reduced Rayleigh scattering attenuation coefficient, as described by Equation (2). An overall reduction in the plume will also minimize any refraction that occurs, as measured by Kataymama et al. [54].

**Figure 6:**
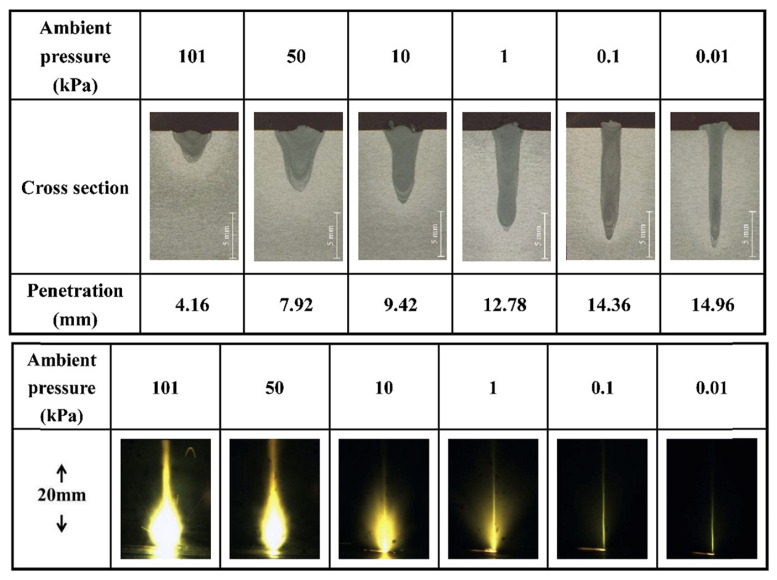
The effects of reducing pressure from ambient at 101 kPa to 0.01 kPa on Yb-fiber welded A5083 aluminum. As pressure is decreased, plume formation is reduced and penetration depth is increased. Reproduced with permission from [56]; 2017, Elsevier.

**Figure 7:**
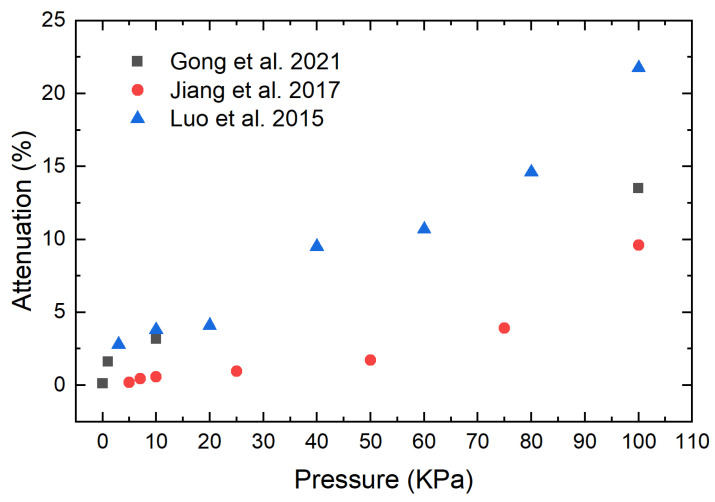
Effect of pressure on attenuation [19,56,59].

The effects of sub-atmospheric processing greatly expand the processing window for laser beam welding, as demonstrated by Jiang et al. [55]. Jiang et al. [56] demonstrated this effect, as shown in Figure 6. It can clearly be seen that—at a reduced pressure—plasma plume generation is significantly reduced and melt depths are increased. Lou et al. [57] also observed reduced plasma plume generation under sub-atmospheric pressure during Yb-fiber welding of 16Mn steel. Chen et al. [58] showed that there is a threshold pressure below which the absorption and scattering effects of the plasma plume are greatly reduced, resulting in deep, high-aspect ratio melt pools of 16Mn steel. For the study performed by Chen et al., this threshold was between 20 and 10 kPa. Lou et al. [59] also observed a critical threshold between 20 and 10 kPa for Yb-fiber welding of 16Mn Steel. Additionally, Lou et al. measured Yb-fiber laser attenuation to be on average 23% at 101 kPa and only 2% at 3 kPa. Through spectrographic analysis of plasma emissions, Jiang et al. [56] confirmed that both electron temperature and electron density are reduced during sub-atmospheric pressure Yb-fiber laser beam welding of A5083 aluminum, with laser attenuation values similar to those observed by Lou et al. in their processing of 16Mn steel. Lou et al. [59], Jiang et al. [56], and Gong et al. [19] all measured laser attenuation through the plasma plume at varying pressures. A summary of the results of these studies is plotted in Figure 7. Here, it can be seen that at atmospheric pressure, the total attenuation is measured to be between approximately 11% and 23%. As pressure decreases, the overall attenuation is greatly reduced to below 5% for all three studies at 20 kPa. Jiang et al. and Gong et al. [19] noted that, in addition to absorption and scattering attenuation, refraction of the laser beam through the plasma plume can also occur, causing the laser beam to shift at the work surface. By reducing the cover gas pressure, Gong et al. measured a decrease in this refraction, resulting in a smaller and more stable effective laser spot.

In addition to the reduced plume leading to decreased beam attenuation, reduced pressure also has the effect of helping to stabilize the keyhole vapor depression. Cai et al. [60] observed reduced porosity in addition to the deeper penetration at reduced pressure. Gao et al. [52] observed melt pools with an in situ X-ray, noting less ejection and attributing this to a more stable keyhole due to weakened metallic vapor ejection. Li et al. [46] and Peng et al. [61] also noted that a reduced difference between melting and boiling temperatures while under vacuum will result in a more stable keyhole. Jiang et al. [62] concluded that a keyhole depression in reduced pressure was more tolerant to vapor pressure variations within the depression, leading to improved stability. Jiang et al. [63] evaluated the flow of the melt pool under reduced pressure and concluded that a more stable fluid flow in the melt pool allows gas bubbles to escape, leading to the noted reduction in porosity. The experimental work of Li et al. [64] concluded that sub-atmospheric pressure reduced the boiling point, leading to a cooler melt pool with more stable fluid convection. Rominger et al. [65] noted these effects (at reduced pressure) also led to a reduction in spatter generated during the welding process. Due to the aforementioned effects of pressure reduction, several authors have demonstrated improved laser beam weld performance in application. Sokolov et al. [66] produced 40 mm thick welds in SM400A steel at 0.1 kPa pressure and Francis et al. [67] produced 80 mm thick welds in SA608 steel at 1.0 kPa pressure, with both rivaling the quality of an electron beam weld.

### 4.2. Hyperbaric

**Figure 8:**
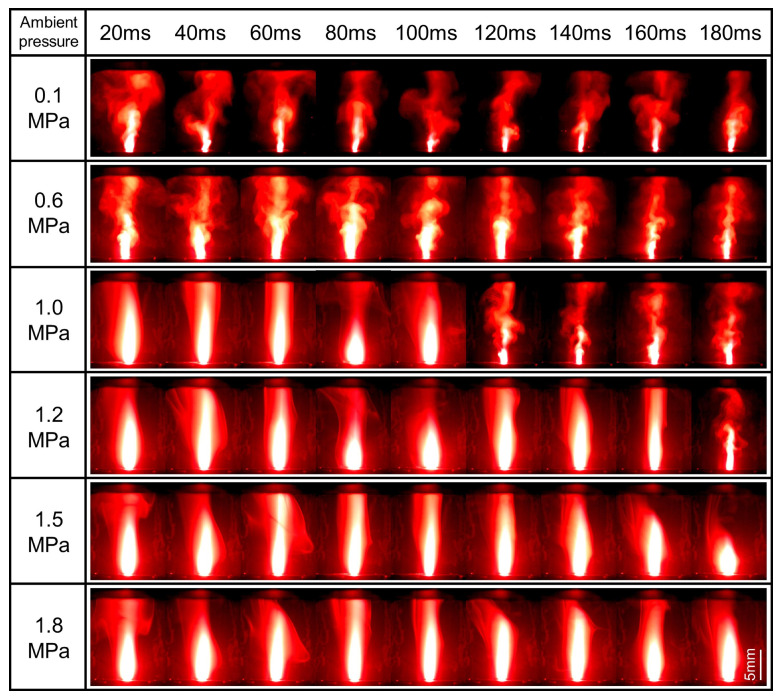
Transient effects of hyperbaric pressures ranging from 0.1 MPa to 1.8 MPa on plasma plume generation in Yb-fiber welding of TA1 titanium from 20 ms after laser exposure to 180 ms after laser exposure. In addition to increased plume intensity with additional pressure, the duration of the plume increases as well. Reproduced with permission from [72]; 2021, Elsevier.

While not as widely researched as sub-atmospheric pressure processing, laser beam welding at hyperbaric pressures has also been researched. Shannon et al. [68] evaluated CO_2_ laser beam welding at pressures ranging from 101 kPa to 5000 kPa. As pressure increased, the size of the plasma plume resulted in increased beam attenuation. It was also noted that—with the increased pressure—the melt pool transitioned from a keyhole mode at atmospheric pressure to a conduction mode at elevated pressures. Several authors have investigated Yb-fiber spot welding of various materials at hyperbaric pressures. Long et al. evaluated spot welding of molybdenum [69], 304L [70], zirconium [71], and TA1 titanium [72]. All of these studies involved using argon at pressures up to 1800 kPa with a Yb-fiber laser. They observed an increase in plasma plume intensity, leading to greater laser beam attenuation and resulting in shallower melt pools. Figure 8 shows the results from Long et al. for titanium. Here, it can be observed that with increasing pressure, plume formation is significantly increased and longer lasting. Su et al. [73] evaluated the microstructure of hyperbaric laser beam welds on 304 stainless steel. In addition to the aforementioned decreased penetration depth, dendrite arm spacing and overall grain size were reduced with increasing pressure. This was attributed to a higher cooling rate due to the increased boiling temperature of 304 stainless steel in the hyperbaric environment. While increased plasma plume generation—as previously discussed—is not desirable, hyperbaric environments enable the processing of high vapor-pressure materials.

### 4.3. Section Summary and Impact on LPBF

The pressure of the atmosphere used can influence all three primary laser beam attenuation mechanisms. In vacuum processing, attenuation is greatly decreased, resulting in more laser power being delivered to the work surface. Vacuum processing also provides an additional benefit in stabilizing the vapor cavity, allowing for very deep melt pools to form without the introduction of porosity through stabilization of the vapor cavity. In hyperbaric processing, attenuation is increased, resulting in less laser power being delivered; however, in limited applications where high vapor-pressure materials are being processed, this is an acceptable trade-off.

The primary benefits to LPBF from pressure manipulation include the potential to reduce porosity through more stable melt pools and increase the efficiency of the laser power being delivered. These attributes enable an expansion of the processing window.

## 5. Current Applications in LPBF

The results presented in Section 3 and Section 4 demonstrate the utility of atmosphere control in varying laser beam welding processes and manipulating material characteristics. While not as widespread as applications in laser beam welding, some authors have explored the various effects of cover gas composition and pressure as they apply to LPBF.

### 5.1. Atmosphere Composition Effects in LPBF

The composition of the cover gas, as presented in Section 3, can have a wide range of possible effects. With the use of a cover gas in laser beam welding, the purpose of the cover gas in LPBF is to inert the atmosphere immediately adjacent to the melt pool to prevent oxidation as well as remove condensate and spatter away from the path of the laser beam. In commercial LPBF, high-purity argon is the most commonly used gas for this purpose and is predominantly used as the baseline for comparison in atmosphere composition research.

Similar to laser beam welding applications, the reactivity of nitrogen can lead to interactions with the base materials. In the fabrication of 17-4PH by LPBF, it has been demonstrated by Murr et al. [74] and Rafi et al. [75] that high amounts of nitrogen in martensitic 17-4PH will result in retained austenite. The effect of this microstructure results in significant variation in mechanical properties [76] and heat treatment performance [77] between the martensitic and austenitic 17-4PH. The source of the nitrogen causing this effect in LPBF 17-4PH is primarily the result of the nitrogen gas used to produce the powder material [74]; however, it has been shown that additional absorption can occur during the LPBF process when nitrogen cover gas is used. Meredith et al. [78] demonstrated that nitrogen can be absorbed from a nitrogen cover gas during continuous wave LPBF, and at a lower magnitude, Brown et al. [76] demonstrated this effect with pulsed wave laser LPBF. In both cases, the amount of absorbed nitrogen was dependent on the processing parameters.

In addition to the well-documented austenite phase stabilization in 17-4PH, the following studies demonstrate the sensitivity of LPBF Ti6Al4V to the cover gas composition. Although well understood that titanium processed by any method is sensitive to oxygen and nitrogen, LPBF provides a unique ability to control absorption. Dietrich et al. [79] measured increased tensile strength and reduced fatigue resistance for LPBF Ti6Al4V with elevated oxygen up to 1000 ppm in an argon atmosphere. Luzon et al. [80] also observed that the presence of reactive oxygen and nitrogen at low concentrations in the process cover gas led to absorption into LPBF Ti6Al4V, causing increased strength and reduced ductility. Chen et al. [81] presented results showing that substituting 25 percent by volume of nitrogen into the argon cover gas led to increased mechanical strength through microstructure refinement, solid solution strengthening, and increased dislocation density.

While Ti6Al4V and 17-4PH are sensitive to cover gas compositions, not all materials are consistently affected. In 316L, Pauzon et al. [82] did not observe any significant microstructure or mechanical performance differences between argon and nitrogen cover gas. Mirz et al. [83] measured a similar indifference in mechanical properties when swapping argon and nitrogen cover gas for 318LN duplex stainless steel. However, Mirz et al. measured a slight increase in irregular porosity and retained austenite for the material fabricated under nitrogen. Bean et al. [84] observed focal point shifts and melt pool shape differences in 718 Inconel when using nitrogen and argon cover gases. These differences were attributed to changes in laser beam attenuation with the two gases.

**Figure 9:**
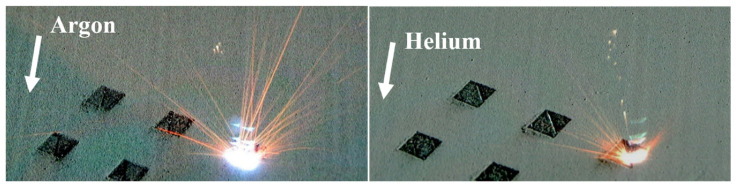
Reduced plume and spatter in LPBF of Ti6Al4V through the use of helium cover gas compared to argon. Reproduced with permission from [87]; 2021, Elsevier.

The differences in argon and nitrogen tend to be material-specific, whereas the effects of helium have been shown to be nearly universal. Pauzon et al. [85], when comparing the effects of helium gas to the more common argon and nitrogen, observed higher strengths and finer microstructures in LPBF 316L. Cabellero et al. [7] compared the effects of helium to argon and observed reduced plasma and a more stable melt pool under helium. Traore et al. [86] also observed reduced plume formation under helium cover gas compared to argon while also noting an increased powder-denuded zone. Pauzon [87] found that the use of helium cover gas reduced the quantity of generated spatter as well as increased cooling rate compared to argon. Images of these findings are found in Figure 9. Here, it can be seen that the intensity of plume formation decreased, and visually, these results are similar to those shown in Figure 4. In addition to the reduced plume intensity, it was also demonstrated that the use of helium reduces the overall spatter generation as well.

### 5.2. Atmosphere Pressure Effects in LPBF

Similar to the effects seen in the literature on laser beam welding, the pressure at which a laser-based process is performed can also have a large effect on melt pool characteristics. There are currently no commercially available pressure-controlled LPBF systems and researchers have built custom machines to explore this area as presented in this section.

**Figure 10:**
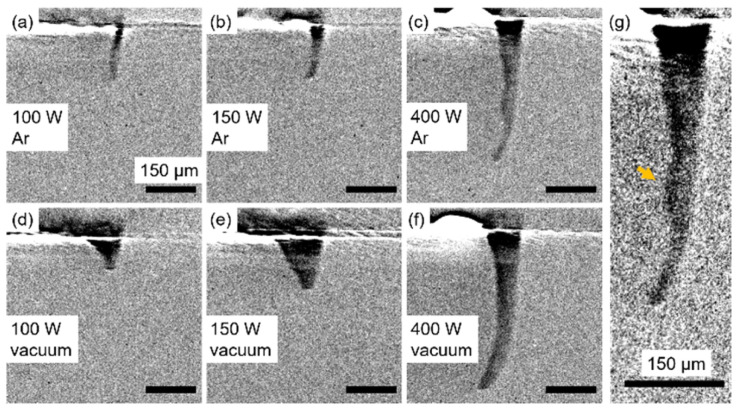
Keyhole vapor depression differences between ambient pressure argon and high vacuum ($1.3\times 10^{-3}$ pa) environments in LPBF 316L at varying laser powers. Reproduced with permission from [88]; 2020, Elsevier.

In laser beam welding, the reduction in ambient pressure increases the stability of the keyhole depression in a melt pool, and this same effect has been observed in LPBF. Through in situ high-energy X-ray imaging, Guo et al. [13] and Calta et al. [88] observed improved keyhole depression stability under sub-atmospheric pressures. Figure 10 shows the effects observed by Calta et al. when comparing the keyhole shape in 316L at various laser powers between ambient and high vacuum environments of $1.3\times 10^{-3}$ pa. In ambient pressure, keyhole instabilities are demonstrated that are not observed when under a vacuum environment.

**Figure 11:**
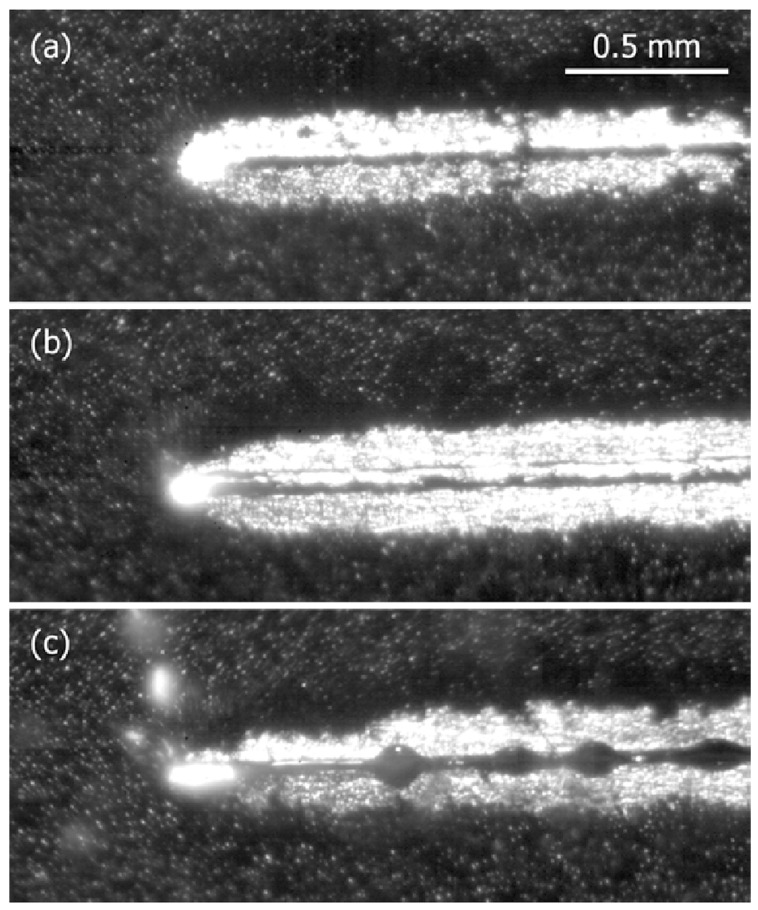
Demonstration of powder denuding at various laser scan parameters for LPBF 316L under 0.1 kPa pressure. Laser power and scan speeds of (**a**) 50 W and 0.2 m/s, (**b**) 100 W and 0.4 m/s, and (**c**) 200 W and 0.8 m/s. Reproduced with permission from [90]; 2018, Elsevier.

When the powder is introduced, however, additional complexity not seen in laser beam welding arises. Matthews et al. [89] demonstrated significant powder denuding, where the pressure differential at the melt pool blew away loose powder, starving subsequent laser scans of powder. Guo et al. [13] also measured this effect in situ, demonstrating that pressure must be tuned to minimize the denuding effect. Bidare et al. [90] produced single-layer scans in a vacuum LPBF system and also observed powder denuding. An example of this denuding effect is shown in Figure 11. As a result, it was suggested that some sort of pre-melt sintering would be required to perform LPBF under vacuum. Achee et at. [91] and Annovazzi et al. [92] both exhibited the feasibility of this technique to enable vacuum processing. Kaserer et al. [93] evaluated the denuding effect in vacuum LPBF and found that the amount of powder denuding was dependent on the working pressure as well as the density of the powder being scanned. It was also demonstrated that for steel and titanium powders, a processing range between 1 kPa and 20 kPa is ideal, as the benefits of vacuum processing were presented without excessive powder movement.

Bidare et al. [94] explored hyperbaric environments and observed increased plasma plume formation at higher pressures, although this could be mitigated by using helium gas instead of argon. Over the experimental window, however, no appreciable process improvements were seen. Griggs et al. [95] demonstrated similar results for single-track experiments, noting that a high-pressure environment could lead to the expanded processing space for material with high melt vapor pressure, similar to applications seen in laser beam welding.

## 6. Conclusions and Outlook

In this review, we presented the current state of the art in utilizing atmospheric variables for optimization in laser-based manufacturing processes, with a special focus on applications in LPBF. The composition of the atmosphere has been shown to have the ability to reduce laser beam attenuation, influence melt pool shape, reduce porosity, change mechanical performance, and manipulate the phase composition of the final material. The manipulation of atmosphere pressure has been shown to reduce laser beam attenuation at the work surface, stabilize the vapor depression in the melt pool, and assist in the processing of high vapor-pressure materials. Combined, the presented literature demonstrates the ability of atmosphere manipulation to provide additional process control, not only in laser beam welding but also in laser-based AM, such as LPBF.

In the context of improving the LPBF process, the ability to reduce instances of keyhole porosity and lack of fusion voids through atmospheric control offers the potential for LPBF hardware to be used in defect-sensitive, high-consequence applications. Additional control also enables an expanded processing window, allowing for novel regimes of process parameter combinations when higher powers are delivered to the work surface. With higher and more stable laser power delivery under helium or vacuum, faster laser scan speeds could be taken advantage of for improved build efficiency. It has also been demonstrated that the use of reactive gases can lead to mechanical performance differences. For the processing of materials sensitive to interstitial strengthening, the addition of nitrogen or carbon dioxide/oxygen could provide additional material performance.

For full adoption of atmosphere manipulation as a standard LPBF critical build parameter, the following barriers need to be addressed:

- Custom equipment is required for atmosphere control, as commercially available LPBF machines currently do not offer the necessary features such as vacuum chambers or gas mixers. Despite this, researchers have begun to apply these principles, exhibiting the potential benefits.
- The nature of the LPBF process—with a free-flowing powder bed—requires powder bed conditioning before vacuum processing can occur. This was demonstrated in a limited laboratory capacity, but maturation is still required to put this into practice.
- The use of vacuum processing shows great potential; however, practical effects of condensate and spatter removal from the laser beam’s scan path under a sub-atmospheric environment have not yet been demonstrated.
- Overall, there is a strong outlook for the adoption of atmosphere manipulation in industrial LPBF applications, pending the resolution of the previous points.

## Figures and Tables

**Table 1:** Common cover gases and their properties.

Properties	Argon	Nitrogen	Helium	Carbon Dioxide
Density (kg·m^−3^)	1.62	1.14	0.16	1.80
Ionization Energy (eV)	15.7	14.5	24.6	13.8
Disassociation Energy (eV)	-	9.756	-	CO_2_ → CO + O @ 5.5
				CO → C + O @ 10.0
				CO_2_ → C + O + O @ 15.5
Thermal Conductivity (W·m^−1^·K^−1^)	0.0178	0.0260	0.156	0.0168
Heat Capacity C*_p_* (J·mol^−1^·K^−1^)	20.83	29.17	20.79	37.52
Chemical Activity	Inert	Reactive	Inert	Oxidizing

Thermophysical properties at 300 K and 101 kPa [15,16,17,18].

## Data Availability

No new data were created or analyzed in this study. Data sharing is not applicable to this article.

## Nomenclature

The following nomenclature abbreviations are used in this review:

AM	additive manufacturing
LPBF	laser powder bed fusion

EBPBF	electron beam powder bed fusion
LDED	laser-directed energy deposition
λ	laser wavelength
$K_{a}$	inverse bremsstrahlung absorption coefficient
$K_{sca}$	Rayleigh scattering coefficient
$n_{e}$	electron number density
$n_{i}$	ion number density
*z*	charge number
*e*	electronic charge
*C*	velocity of light
$\epsilon_{0}$	dielectric constant of medium
$m_{e}$	mass of electron
$k_{B}$	Boltzmann constant
*T*	temperature
ω	angular frequency of a laser beam
$\omega_{pe}$	angular frequency of plasma oscillation
lnΛ	Coulomb logarithm
$K_{sca}$	Rayleigh scattering coefficient
*N*	number density of particle
*V*	particle volume
ϵ	dielectric constant of particle
$T_{e}$	electron temperature
